# Characterizing the Core Internal Gene Pool of H9N2 Responsible for Continuous Reassortment With Other Influenza A Viruses

**DOI:** 10.3389/fmicb.2021.751142

**Published:** 2021-12-16

**Authors:** Haoyi Yang, Mingda Hu, Boqian Wang, Yuan Jin, Xingfei Gong, Long Liang, Junjie Yue, Wei Chen, Hongguang Ren

**Affiliations:** ^1^Beijing Institute of Biotechnology, State Key Laboratory of Pathogen and Biosecurity, Academy of Military Medical Sciences, Beijing, China; ^2^College of Computer, National University of Defense Technology, Changsha, China

**Keywords:** H9N2, reassortment, influenza A virus, evolution, genome

## Abstract

Reassortment among avian influenza viruses is the main source of novel avian influenza virus subtypes. Studies have shown that the H9N2 virus often donates internal segments to generate novel reassortant avian influenza viruses, acting as a reassortment template. However, the characteristics of the internal pattern of reassortment remain unclear. In this article, we first defined the core gene pool of the internal segments of the H9N2 virus that provide templates for reassortment. We used genetic distance and sequence similarity to define typical clusters in the core gene pool. Then, we analyzed the phylogenetic relationships, feature vector distances, geographic distributions and mutation sites of strains related to the core gene pool. Strains in the same typical clusters have close phylogenetic relationships and feature vector distances. We also found that these typical clusters can be divided into three categories according to their main geographic distribution area. Furthermore, typical clusters in the same geographic area contain some common mutation patterns. Our results suggest that typical clusters in the core gene pool affect the reassortment events of the H9N2 virus in many respects, such as geographic distribution and amino acid mutation sites.

## Introduction

H9N2 avian influenza virus (AIV) was first detected in 1966 (Homme and Easterday, 1970). Since then, it has been found to be widespread in avian around the world, especially in the last two decades (Peacock et al., 2019). H9N2 infects hosts and transmits rapidly, as its low pathogenicity gives it high fitness in poultry and other wild birds. In a recent report, H9N2 was found to be the dominant subtype of AIVs among poultry in China (Bi et al., 2020). The first zoonotic event involving H9N2 was reported in 1997 (Peiris et al., 1999). Since then, several cross-species transmissions to humans or swine have been documented (Sun et al., 2020).

A number of AIVs have been reported to infect humans, including H7N9, H5N6, H10N8, H5N1, H6N1, and H7N4 (Guan et al., 1999; Hoffmann et al., 2000; Gao et al., 2013; Chen et al., 2014; Yang et al., 2015; Li et al., 2020; Qu et al., 2020). Some of these human-infecting AIVs [e.g., H7N9, H5N1, H10N8, and H5N6 (Guan et al., 1999; Martin et al., 2011; Chen et al., 2014; Cui et al., 2014; Yang et al., 2015; Pu et al., 2021)] were found to be reassortant viruses, with H9N2 contributing the internal segments to the reassortants (Liu et al., 2014). Hosts may be coinfected with H9N2 and other AIV subtypes, and in this context, H9N2 may contribute reassortment templates for novel reassortment AIV subtypes. The frequent reassortments among the H9N2 virus and other subtypes of AIVs imply that there may be a core internal gene pool in the H9N2 virus that continuously offers segments for the emergence of novel AIVs.

In this article, we aimed to identify and characterize the core gene pool of H9N2 internal segments and analyze the reassortment events involving the H9N2 virus. Based on a genetic analysis and mathematical relationships calculated for all the H9N2 viruses and other relevant AIVs, we defined typical clusters of each internal segment of the H9N2 virus. Each typical cluster consists of strains that are clustered together according to sequence similarity and evolutionary tree branching information. Strains in the same typical cluster have similar genetic characteristics. Typical clusters with different characteristics constitute the core gene pool of H9N2 internal segments. Then, we extracted feature vectors from the sequences and performed mathematical clustering to cross-validate the results. The clustering results essentially corresponded to the typical clusters. Then, we conducted a more detailed analysis of the biological characteristics of the core gene pool. The results reflect that different typical clusters infect different host species and have different geographic distributions. Mutation site analysis of typical clusters revealed that typical clusters in the same geographic area share some common mutation sites. We further analyzed the relationship between H7N9 and H9N2 using mathematical feature characterization. Most sequences of the H7N9 virus and H9N2 virus clustered together, which is consistent with reported reassortment events and further verifies the rationality of our core H9N2 virus gene pool.

## Materials and Methods

### Sequence Data Preparation

All internal segments sequences of H9N2 virus and other relevant AIV subtypes are downloaded from NCBI Influenza Virus Resource (Bao et al., 2008). Only full-length sequences are preserved. Repetitive sequences with the same host, the same country, the same time and the same subtypes are excluded. For the relevant AIVs other than H9N2, sequences with >98% similarity to H9N2 virus are selected judged by blast (Johnson et al., 2008). MAFFT (Katoh and Standley, 2013) are used to align the coding region of the resulting sequences and then Mega (Kumar et al., 2016) is used to manually inspect the sequences. The sequence lengths of different AIV subtypes are slightly different. So, we set a lower threshold for each internal segment sequences, as Table 1 shows. For MP and NS segments, M1 and NS1 segment are considered in the follow-up study.

**TABLE 1:** Lower threshold for internal segments nucleotide sequence length.

Internal segments	PB2	PB1	PA	NP	MP	NS
Sequence length	2280	2274	2151	1497	759	693

We use CD-HIT to initially filter the resulting sequences with a threshold level of 0.98 and retained sequences in clusters which meet all the following rules.

• Cluster contains H9N2 virus sequence(s).
• Besides H9N2 virus sequence(s), cluster contains other AIV subtypes sequences. And there is at least one sequence whose collecting time is no more than 3 years from the collecting time of H9N2 virus sequence(s) in the cluster.
• Besides H9N2 virus sequence(s), cluster contains other AIV subtypes sequences. And there is at least one sequence which has the same host or country with H9N2 virus sequence(s).

After first filtering, our dataset includes 2,583 PB2 sequences, 3,356 PB1 sequences, 3,565 PA sequences, 3,660 NP sequences, 5,757 MP sequences, and 3,325 NS sequences.

### Phylogenetic Tree Construction and Analysis

Maximum-likelihood trees are inferred by IQ-TREE (Nguyen et al., 2015). And ModelFinder is used to find the best partition model automatically (Kalyaanamoorthy et al., 2017). Branch supports are obtained with 1,000 times ultrafast bootstrap (Hoang et al., 2018). Both ModelFinder and the ultrafast bootstrap are implemented in IQ-TREE.

For each evolutionary tree, we use Mean Pairwise Distance (MPD) to cluster AIV strains (Tsirogiannis and Sandel, 2014). The MPD formula is as follows:


$$m\,p\,d\,=\frac{\sum_{\text{i}}^{\text{n}}\sum_{\text{j}}^{\text{n}}\delta_{i,j}}{\left(\begin{array}{c} \text{n}\\ 2 \end{array}\right)}$$

We calculate the median number of leaf nodes and MPD for each internal node. To reduce deviation, we also set a lower threshold for the median number of leaf nodes. We choose the larger one of the median and the threshold as the max number of strains in a cluster, named as *maxNode*. The average MPD of all internal nodes is defined as *meanMPD*.

### Definition of Typical Clusters

When two different strains have a reassortment relationship, we believe that their sequences are similar and they have close phylogenetic distance reflected in the evolutionary tree. For a given subtree *s* of the evolutionary tree, it has two features. *N*_*s*_ reflects the number of strains of subtree *s*, and *mpd*_*s*_ represents the MPD value of subtree *s*. We define a set K(s) which contains all strains in subtree s. To infer the possible genealogy and relationship of strains, we use the following conditions to measure phylogenetic distance between strains: (a) Strains in subtree s with *N*_*s*_ < *maxNode* have close phylogenetic distance. (b) Strains in subtree s with *mpd*_*s*_ < *meanMPD* have close phylogenetic distance. Subtree that satisfies one of the above conditions is defined as a cluster.

We analyze the common mutation sites for all strains in a given cluster. A cluster with more than one strains that contains H9N2 virus and has common non-neutral mutations is defined as a typical cluster.

### Feature Extraction

To verify the rationality of typical clusters and characterize each genotype completely, we define a feature vector for each genotype. In DNA/RNA sequences, there are 64 combinations of triplet nucleotide residue sequences (codons). Excluding termination codons, 61 codons remain. Each sequence starts from the initiation codon and every three bases is a codon. Bases included in a sequence compose a set *S*. For each base occurs in the sequence, the frequency of it is defined as *p*_*i*_,(*i* ∈ *S*). For each codon except termination codons, the frequency of it can be defined as *p*_*ijk*_,(*i*,*j*,*k* ∈ *S*). Trinucleotide relative abundance (TRA) is an extension of dinucleotide relative abundance (DRA; Kariin and Burge, 1995), which reflects the correlation between three adjacent bases. The TRA of a codon is defined as:


$$T_{i\,j\,k}=\frac{p_{i\,j\,k}}{p_{i}\,p_{j}\,p_{k}}$$

Relative synonymous codon usage (RSCU; Sharp et al., 1986) is used to access codon usage bias. For a given codon, the number of times it appears in the sequence is defined as *Obs*_*codon*. The times that the coded amino acid occurs is defined as *Obs*_*Amino*, while the number of synonymous codons of the amino acid is denoted as *n*. When those synonymous codons don’t have codon usage bias, the expected observation number of them in the sequence is calculated as follows. The RSCU value of a given codon is calculated as follows:


$$E\,x\,p\,\_\,c\,o\,d\,o\,n_{i}=\frac{O\,b\,s\,\_\,A\,m\,i\,n\,o_{i}}{n},c\,o\,n\,d\,o\,n_{i}\in A\,m\,i\,n\,o_{i}$$

$$R\,S\,C\,U_{i}=\frac{O\,b\,s\,\_\,c\,o\,d\,o\,n_{i}}{E\,x\,p\,\_\,c\,o\,d\,o\,n_{i}}$$

Considering both TRA value and RSCU value of codons occur in a sequence, our feature vector is the product of RSCU and TRA:


V=R⁢S⁢C⁢U×T

As three termination codons are excluded, the feature vector is a 61-dimensional vector.

### Core Gene Pool Characterization

A typical cluster in core gene pool must have long time span or contain large number of AIV subtypes or contain large number of strains. We screen qualifying typical clusters from the typical clusters calculated in the previous section. These qualifying clusters have more than 3-year time span, or the number of AIV subtypes or strains they contain is higher than the median value. We calculate the average center of each screened cluster as the center vector, which can characterize the cluster. Then we calculate a distance matrix among all the screened clusters and show it in the form of a heat map. In addition to mathematical characterization, we also use common amino acid mutation sites and geographic distribution to characterize clusters biologically.

### Analysis of Reassortment Events

We find H7N9 virus is the most representative reassortment AIV subtypes which has been reported before. TSNE (Pezzotti et al., 2017) method can reduce the dimensionality of high-dimensional data and map it to two-dimensional or three-dimensional space. To visually show the relationship between H7N9 virus and H9N2 virus, we use TSNE method perform dimensionality reduction visualization on all the feature vectors. Then we mark all H9N2 virus sequences and H7N9 virus sequences in the core gene pool clusters to verify the rationality of our clusters.

## Results

### Phylogenetic Tree Clustering

**FIGURE 1:**
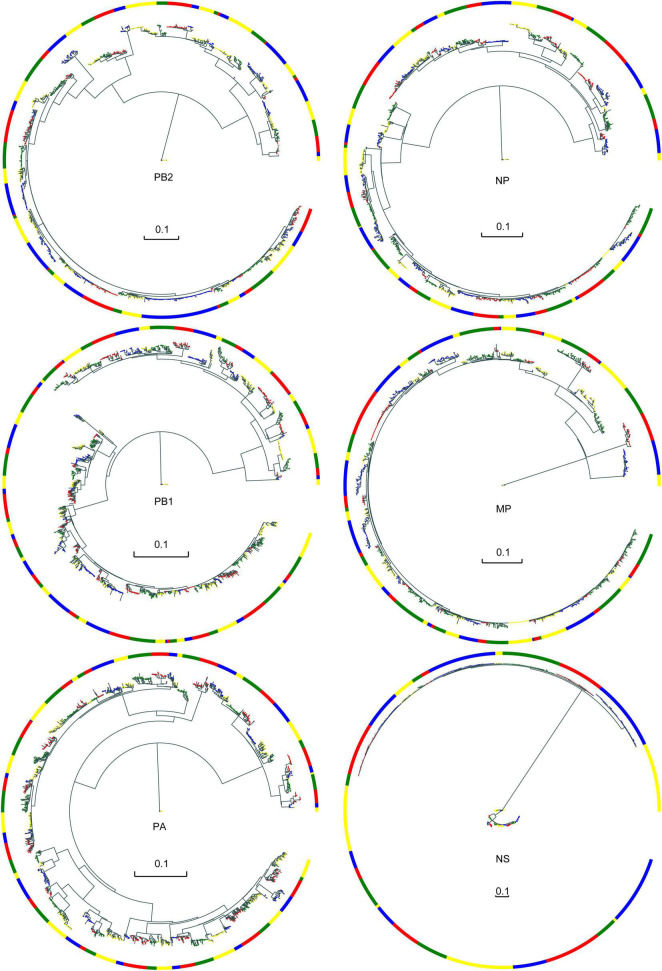
Evolutionary trees and typical clusters of the core gene pool. The branches are colored according to their different typical clusters. Color blocks are visual representations of different typical clusters. Each color block represents a typical cluster; the colors themselves have no special meaning.

We downloaded all available internal segment sequences of AIV from NCBI as of October 13, 2020, and finally constructed a genome set including 1,428 PB2 sequences, 1,795 PB1 sequences, 1,988 PA sequences, 1,564 NP sequences, 1,318 MP sequences, and 637 NS sequences. We constructed six phylogenetic trees for the six internal segments. According to the MPD value and similarity of leaf nodes in the phylogenetic trees, sequences of each internal segment were clustered into different clusters, as shown in Figure 1. The different colors of the blocks are used only to distinguish between the different typical clusters. Although some typical clusters contain a smaller number of strains, their frequency of occurrence (number of subtypes or number of strains occurring in a year) is high. As a result, we also consider them typical clusters. Additional information about the typical clusters of the six internal segments, including host, location distribution, AIV subtypes and so on, is shown in Supplementary Figures 1–6.

Almost all six internal segments had similar numbers of typical clusters and sequences (Table 2). The PB1 and PA segments have a larger number of typical clusters and sequences. Thus, they may be more likely to be reassortment templates.

**TABLE 2:** Numbers of typical clusters for six internal segments.

Internal segments	PB2	PB1	PA	NP	MP	NS
Typical clusters	55	73	65	56	56	26

### Mathematical Characterization of Typical Clusters

**FIGURE 2:**
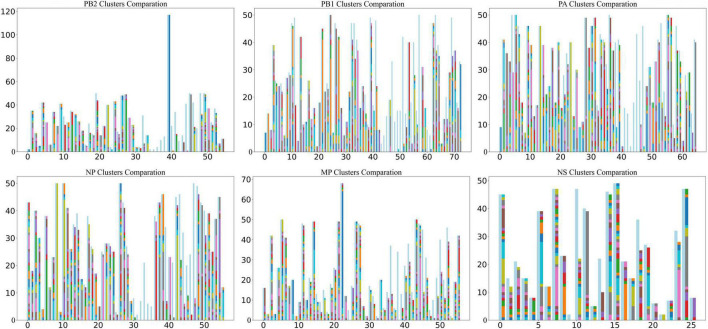
Comparison of BRICH cluster results and typical clusters. The horizontal coordinate is the typical cluster number, and the vertical coordinate is the number of strains included. The light blue bars show the strains included in typical clusters, and the different colored bars on the right are the clusters calculated by the BRICH method. When all strains in a BRICH cluster can be found in a typical cluster, the bar representing the BRICH cluster corresponds to the typical cluster, as shown in the figure.

To cross-validate the correctness of the clustering results and preserve the features of each sequence as completely as possible, we used a 61-dimensional feature vector to represent a sequence. We used BRICH (Zhang et al., 1996) to cluster the sequence feature vectors (Figure 2). The BRICH method clusters strains in terms of vector distance only, making the number of clusters higher than the number of original typical clusters. When all strains in a BRICH cluster can be found in a typical cluster, we consider that the BRICH cluster corresponds to that typical cluster, but the converse is not true. In this analysis, most clusters were observed compared to the phylogenetic clustering method. Figure 2 shows that some of the resulting BRICH clusters cannot correspond to typical clusters. This phenomenon may occur in part because biological significance is neglected in the mathematical characterization. We analyzed these special typical clusters and found that they were basically composed of H9N2 and H7N9 viruses.

**FIGURE 3:**
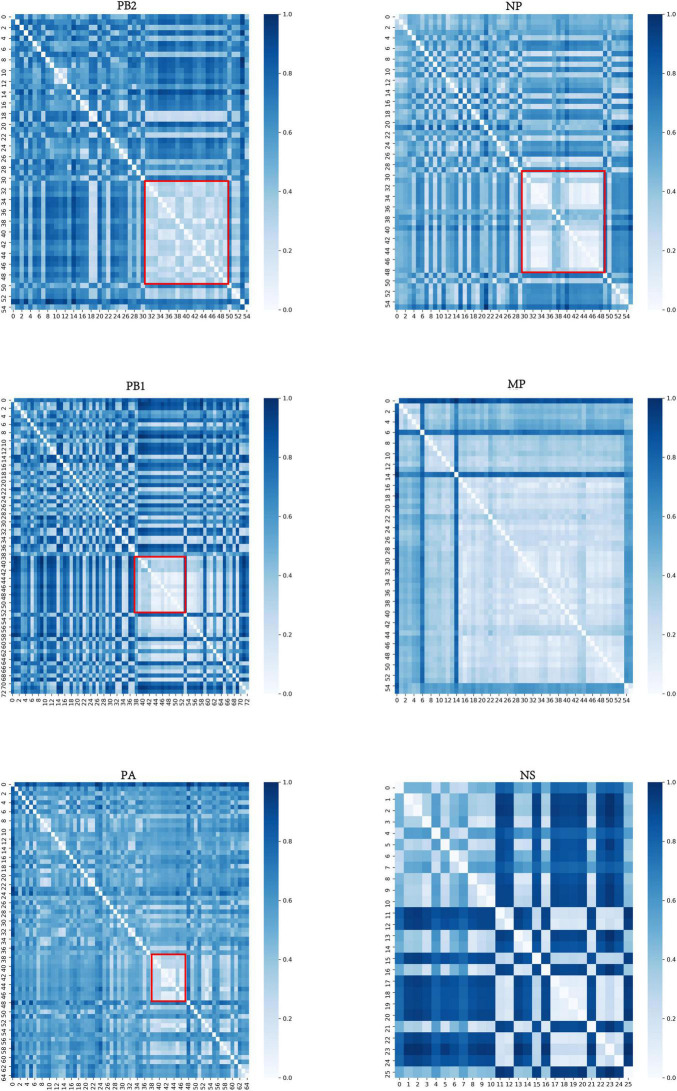
Distance matrix of typical clusters of six internal segments. The horizontal and vertical coordinates represent typical clusters. The color shading corresponds to the distance between typical clusters. The darker the color is, the more distant the corresponding two typical clusters are. The light-colored areas (marked by a red box) indicate that those typical clusters have relatively close distances. The distance matrices of MP and NS segments are not marked as they have few significances.

For each internal segment, we calculated the distance matrix among typical clusters based on the center vector of each typical cluster and then normalized the distance matrix. The resulting heatmap, shown in Figure 3, indicates that there are obvious differences between each typical cluster. The light-colored areas (marked by a red box) indicate typical clusters that have relatively close distances, which is consistent with the special typical clusters mentioned above.

### Biological Characterization of Typical Clusters

**FIGURE 4:**
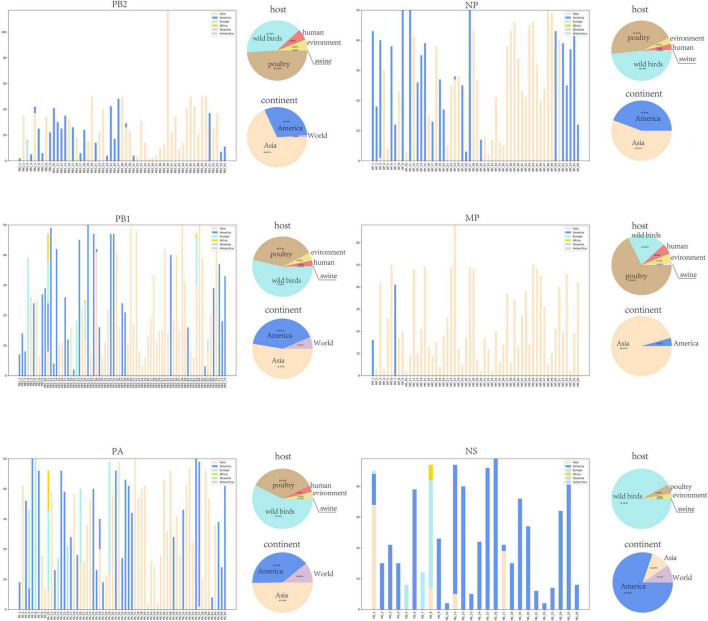
Location and host information for typical clusters. The histogram shows the geographical distribution of each typical cluster and the number of strains contained in corresponding regions. The horizontal coordinate of the histogram is the typical cluster number, and the vertical coordinate is the number of strains contained in different regions. The geographical regions counted in the histogram include Asia, America, Europe, Oceania, and Antarctica. The two pie charts show the host species of all strains and their main geographical distributions. The pie chart of host species counts the proportion of five host species including wild birds, human, poultry, swine, and environment. And the pie chart of geographical distributions counts the proportion of three region types including America, Asia, and World.

Beyond the mathematical characterization of typical clusters in the core gene pool, we are more interested in the biological characterization of these clusters, which can provide more detailed biological insights. We found that there were obvious differences in geographic distribution among these typical clusters in the core gene pool. As shown in Figure 4, the geographical distribution of all typical clusters can be divided into three categories. We named the three categories Asia-Clusters, America-Clusters, and World-Clusters according to the distribution of typical clusters. Of these, Asia-Clusters and America-Clusters account for the majority. The proportions of the three categories in MP and NS segments are different from those in the other four internal segments. In the MP segment, Asia-Clusters account for the majority, while America-Clusters are predominant in the NS segment.

Furthermore, we found relationships between the common mutation sites and geographical distribution of those typical clusters. Taking common mutation sites of the PB2 segment as an example (Table 3), we found that over 80% of the strains with PB2 segments including 477I were found in the Americas. Conversely, we found that over 80% of the strains in the Americas have PB2 segments with 477I. As a result, we can infer rationally that strains with 477I in their PB2 segments are likely to be distributed in or originate from the Americas. The complete relationship between mutation sites and geographic distribution is shown in Supplementary Tables 1–6.

**TABLE 3:** Relationship between mutation sites and geographic distribution of PB2.

Location	Mutation site
America	477I, 647M, 452Q, 291M, 450L
Asia	388K, 647V, 273T, 587V, 196N, 597V, 675V, 423S, 105A, 291V
Europe	18V

### Analysis of H7N9 and H9N2 Virus

**FIGURE 5:**
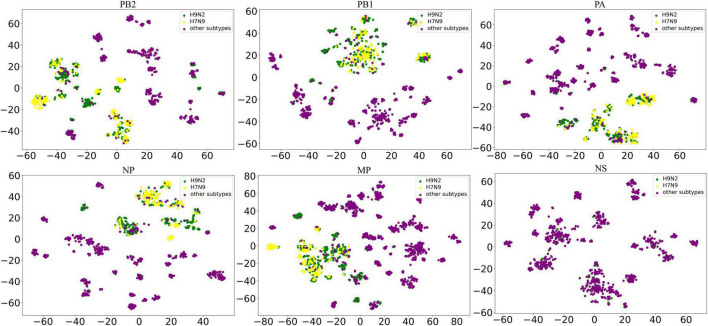
Dimensionality reduction scatter plots of H7N9 and H9N2. The horizontal and vertical coordinates are the values of the horizontal and vertical coordinates corresponding to the downscaling of the sequence feature vector to a two-dimensional space, which has no practical significance. A green dot represents a H9N2 virus, while a yellow dot represents a H7N9 virus. The closer the two virus sequence feature vectors are, the more concentrated the two dots are. We only studied the relationship between H9N2 and H7N9, so other AIV subtypes are represented by dark purple dots.

Among all the reported reassortment events related to the H9N2 virus, the H7N9 virus is highly representative. It is widely distributed and has the ability to infect humans. Notably, it has evolved a lineage with high pathogenicity (Yang et al., 2017). Meanwhile, the H7N9 virus exhibited unique behavior in the above mathematical characterization analysis. As a result, we used the feature vectors of all sequences to construct two-dimensional scatter plots using the TSNE method (Pezzotti et al., 2017). Then, we marked all H9N2 virus sequences and H7N9 virus sequences in the scatter plots (Figure 5). Each scatter represents a sequence. For most segments, the H7N9 virus sequences and H9N2 virus sequences are clustered together in the scatter plots. The close distance indicates that these strains have a close relationship. This can also verify the rationality of the feature vectors we extracted and the core gene pool clustering in this analysis.

## Discussion

**FIGURE 6:**
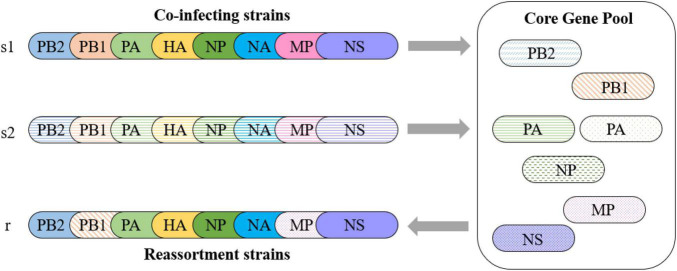
Reassortment pattern of the core gene pool. Two strains, s1 and s2, coinfect a host with the H9N2 virus. Strain s1 obtains PB1 and MP internal segments from the core gene pool and produces a reassortment strain r. Strain s2 provides a new PA for the core gene pool.

The low pathogenicity and wide distribution of the H9N2 virus make it more likely to be coinfected with other AIV subtypes. As explained above, we consider that the internal segments of the H9N2 virus can be regarded as a gene pool that is in a state of dynamic equilibrium. Avian influenza virus coinfected with the H9N2 virus can be regarded as entering the gene pool of the H9N2 virus, where it may not only provide new internal segments for the gene pool but also receive internal segments from the gene pool and generate novel reassortment AIV subtypes (Figure 6). Those internal segments that appear in the gene pool with high frequency constitute the core gene pool of the H9N2 virus.

The establishment of the core gene pool of the H9N2 virus in this article provides new insight into the reassortment characteristics of the H9N2 virus. We conducted systematic phylogenetic analysis and detailed analysis of different characterizations and specific reassortment. The results indicate that the core gene pool of the H9N2 virus affects the evolution direction and geographical distribution of reassortment events related to the H9N2 virus.

The feature vector distance of sequences and the genetic distance of strains show obvious aggregation among strains. Based on the aggregation of different strains, the internal segments of strains can be divided into several typical clusters. The analysis of strain collection locations and collection times indicates that there is geographic isolation among different lineages. The hosts of H9N2 virus and relevant reassortment virus are mainly avian, resulting in rare transmission across continents; this is the main reason for geographic isolation. In total, typical clusters located in Asia and the Americas account for the main part of the core gene pool. The distribution differs among different internal segments. Further amino acid mutation site analysis suggests some valuable mutation sites related to geographical distribution. Finally, the analysis of the H7N9 virus visually demonstrated its reassortment relationship with the H9N2 virus, which also verified the rationality of our core gene pool.

It should be noted that the establishment of the core gene pool depends on the existing sequence data. Although we aimed to exclude the impact of sampling bias on the results as much as possible, it is inevitable that our results will differ from the real situation in some ways. Moreover, the analysis of mutation sites in this article is limited to the strains in the core gene pool. We also conducted an analysis of the mutation sites of all available sequences, which produced completely different results. In summary, the core gene pool in this article can help us understand the pattern in which the H9N2 virus donates internal segments during reassortment with other AIV subtypes. In future work, we will further investigated the impact of the core gene pool on the evolution and fitness of the H9N2 virus.

## Data Availability Statement

The original contributions presented in the study are included in the article/Supplementary Material, further inquiries can be directed to the corresponding author/s.

## Author Contributions

HR, WC, and JY formulated the study. HY and MH performed the research and analyzed the data. BW, YJ, XG, and LL participated in analysis and discussion. HY and HR drafted the manuscript. All authors read and approved the final manuscript.

## Conflict of Interest

The authors declare that the research was conducted in the absence of any commercial or financial relationships that could be construed as a potential conflict of interest.

## Publisher’s Note

All claims expressed in this article are solely those of the authors and do not necessarily represent those of their affiliated organizations, or those of the publisher, the editors and the reviewers. Any product that may be evaluated in this article, or claim that may be made by its manufacturer, is not guaranteed or endorsed by the publisher.

## Supplementary Material

Supplementary Figure 1 Complete information about typical clusters of PB2.

Supplementary Figure 2 Complete information about typical clusters of PB1.

Supplementary Figure 3 Complete information about typical clusters of PA.

Supplementary Figure 4 Complete information about typical clusters of NP.

Supplementary Figure 5 Complete information about typical clusters of MP.

Supplementary Figure 6 Complete information about typical clusters of NS.

Supplementary Table 1 Complete relationship between mutation sites and geographic distribution of PB2. All mutation information was obtained by comparing the filtered sequence data.

Supplementary Table 2 Complete relationship between mutation sites and geographic distribution of PB1. All mutation information was obtained by comparing the filtered sequence data.

Supplementary Table 3 Complete relationship between mutation sites and geographic distribution of PA. All mutation information was obtained by comparing the filtered sequence data.

Supplementary Table 4 Complete relationship between mutation sites and geographic distribution of NP. All mutation information was obtained by comparing the filtered sequence data.

Supplementary Table 5 Complete relationship between mutation sites and geographic distribution of MP. All mutation information was obtained by comparing the filtered sequence data.

Supplementary Table 6 Complete relationship between mutation sites and geographic distribution of NS. All mutation information was obtained by comparing the filtered sequence data.
