# Supplementary figures and images for: Characterizing the Core Internal Gene Pool of H9N2 Responsible for Continuous Reassortment With Other Influenza A Viruses
Source: Front Microbiol. 2021 Dec 16;12:751142. doi: 10.3389/fmicb.2021.751142 (PMC8717948; doi:10.3389/fmicb.2021.751142)

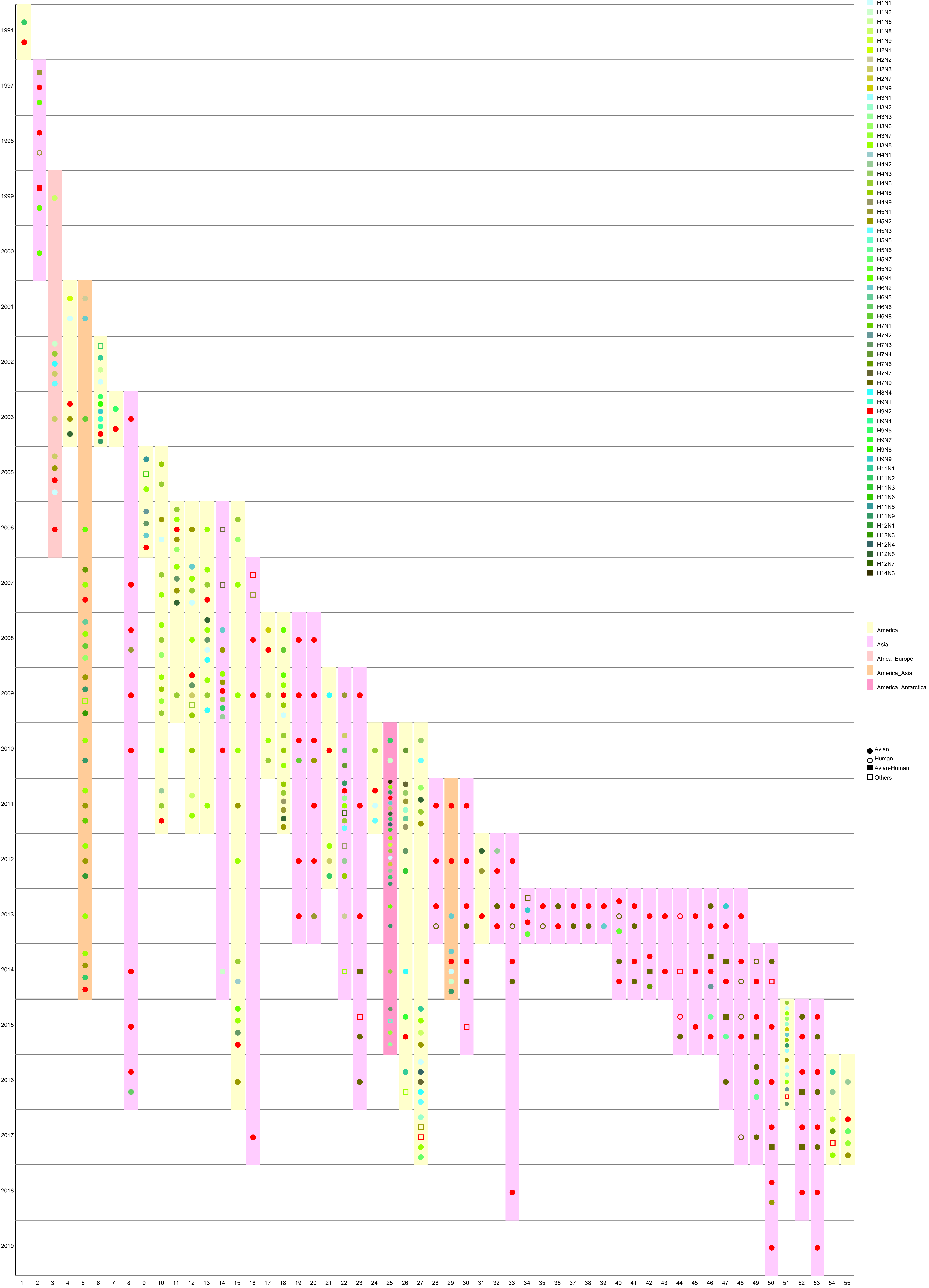

Supplement: Supplementary Figure 1 — Complete information about typical clusters of PB2. [file Image_1.PDF]

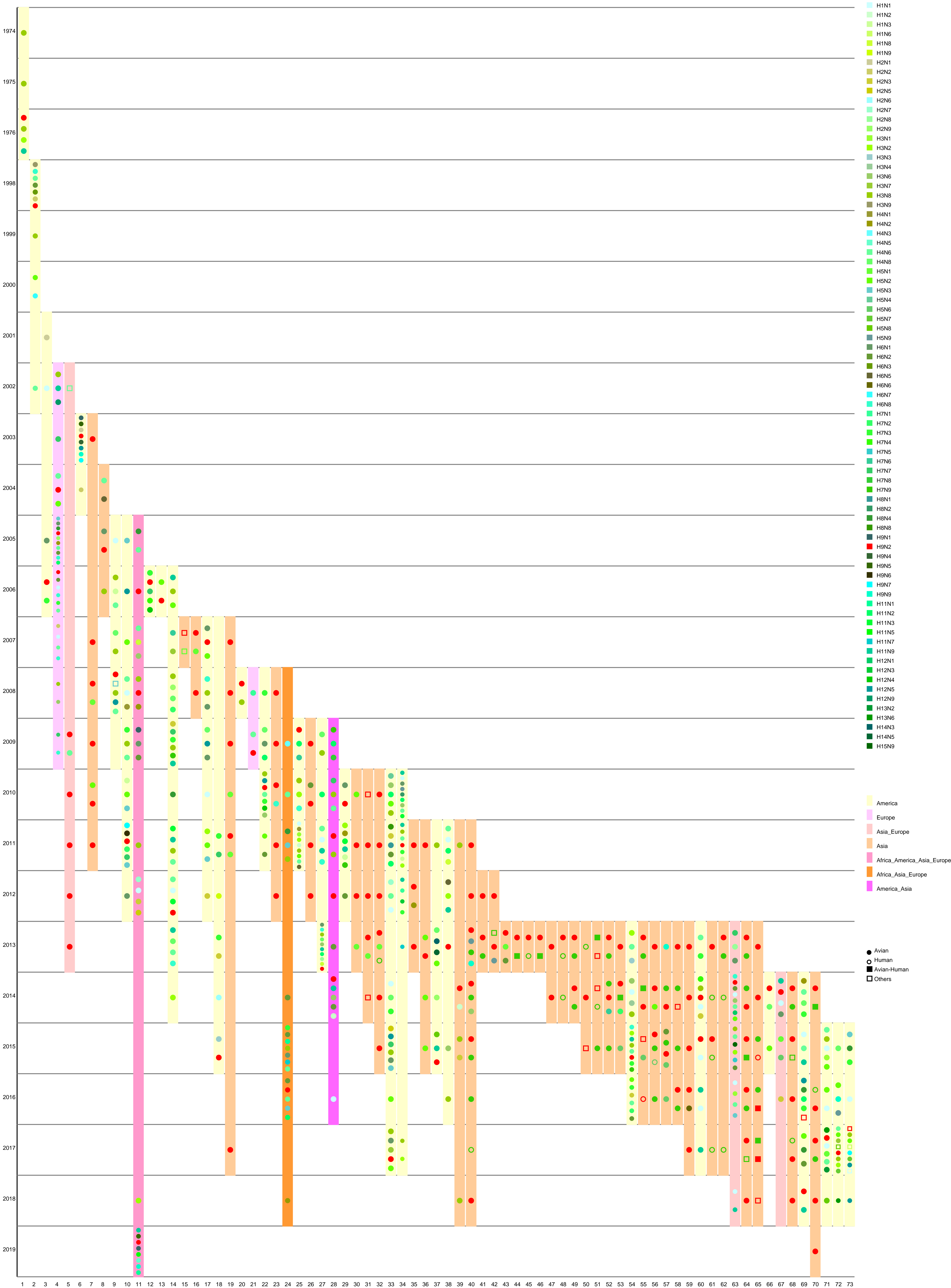

Supplement: Supplementary Figure 2 — Complete information about typical clusters of PB1. [file Image_2.PDF]

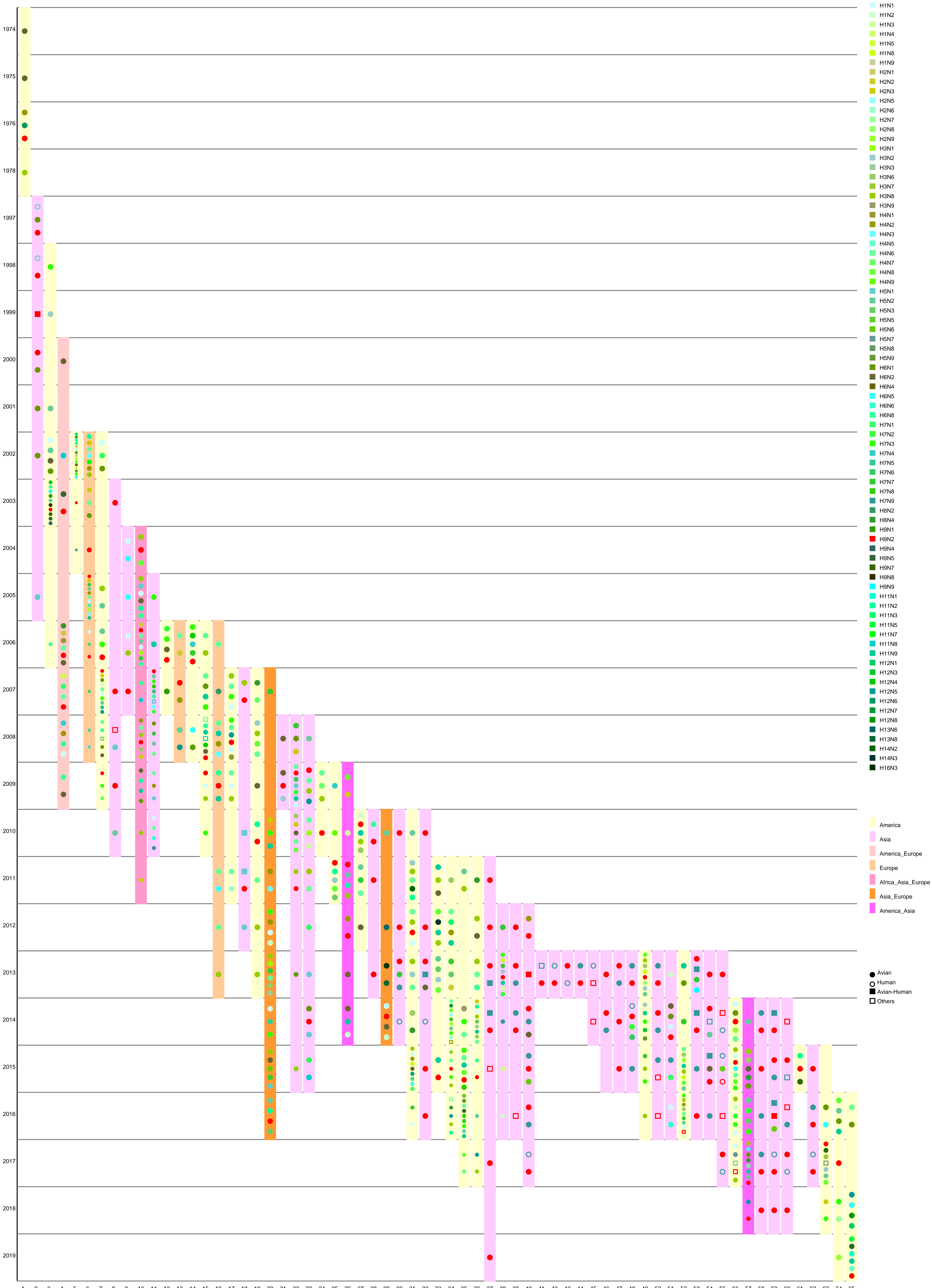

Supplement: Supplementary Figure 3 — Complete information about typical clusters of PA. [file Image_3.PDF]

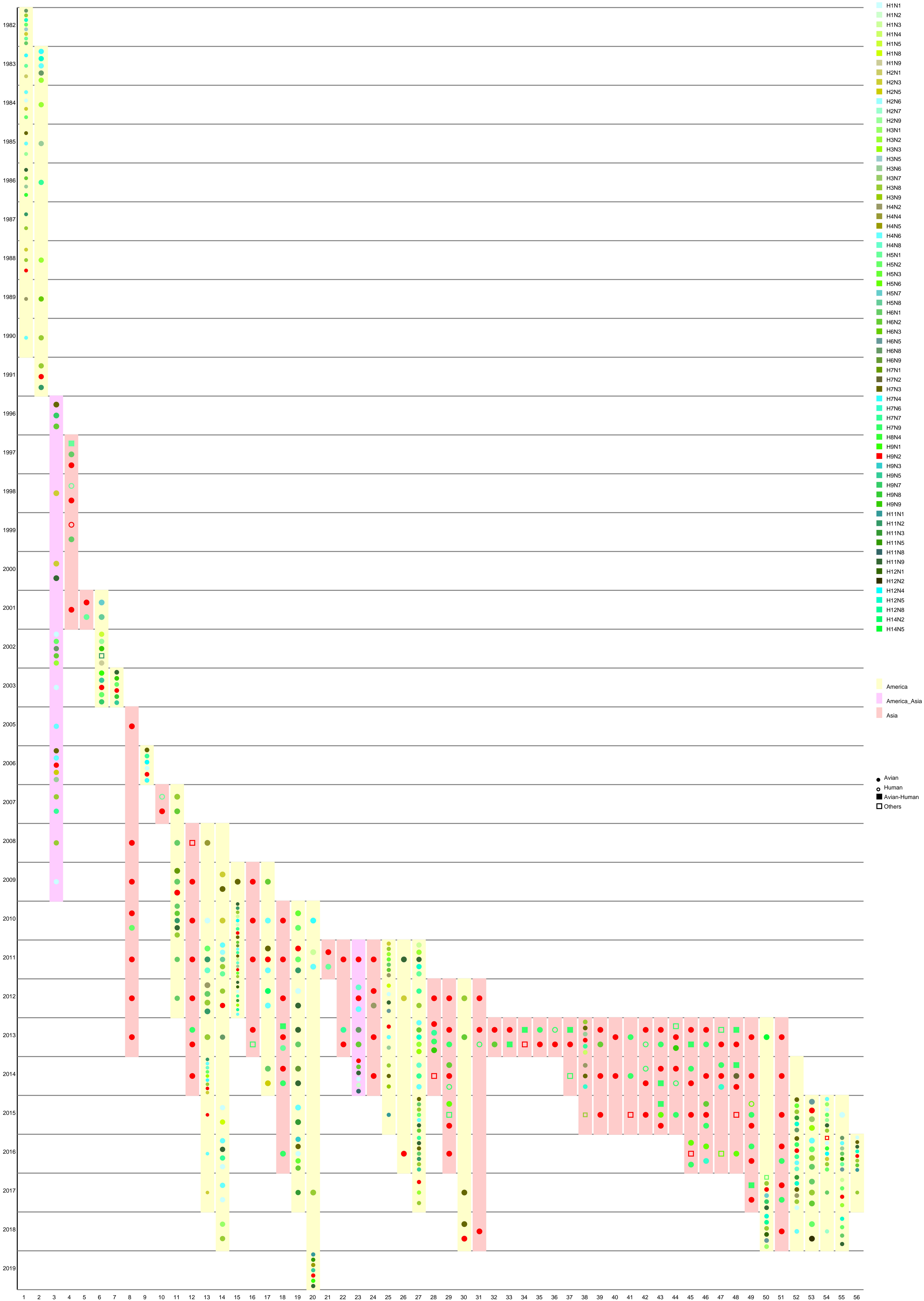

Supplement: Supplementary Figure 4 — Complete information about typical clusters of NP. [file Image_4.PDF]

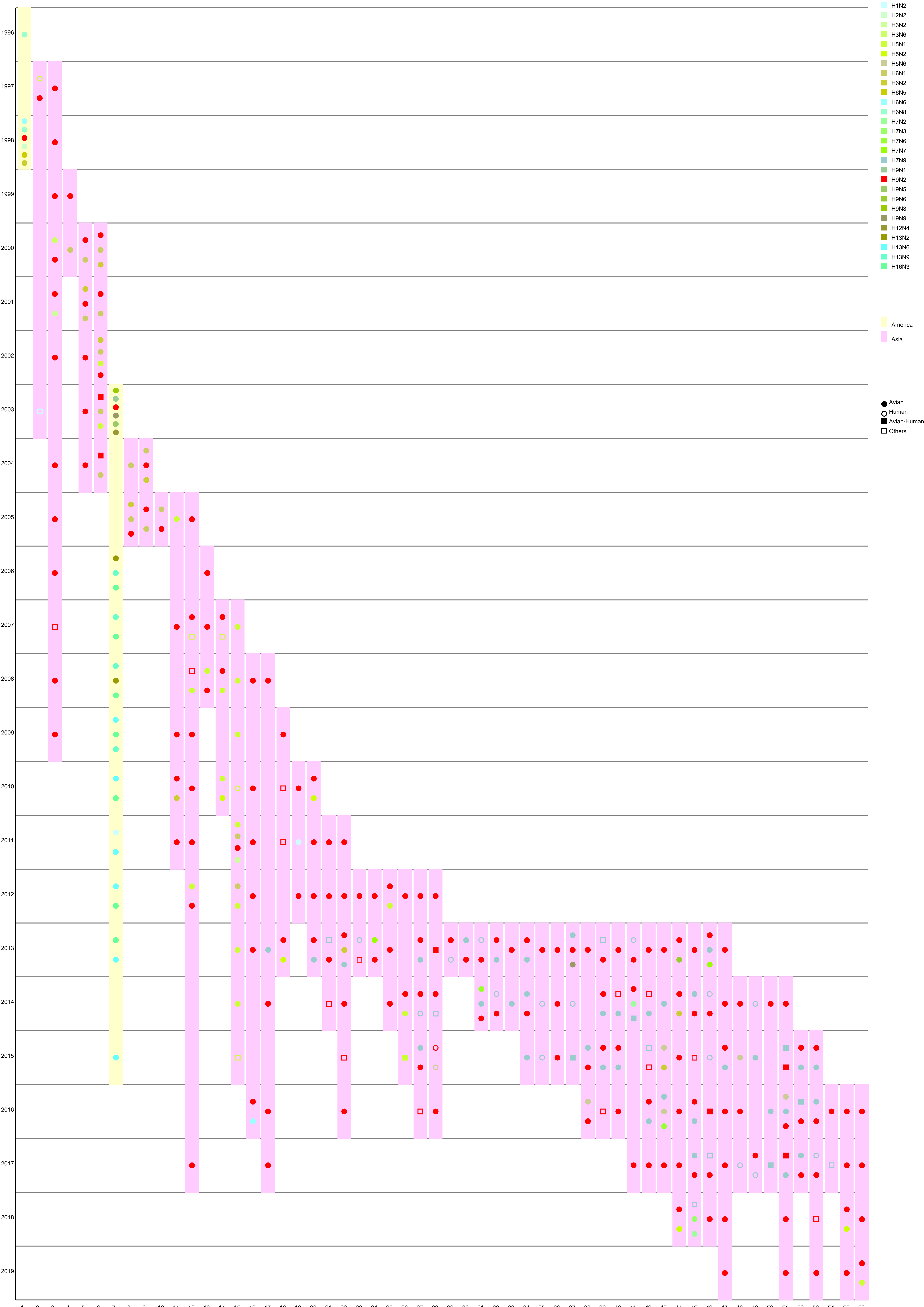

Supplement: Supplementary Figure 5 — Complete information about typical clusters of MP. [file Image_5.PDF]

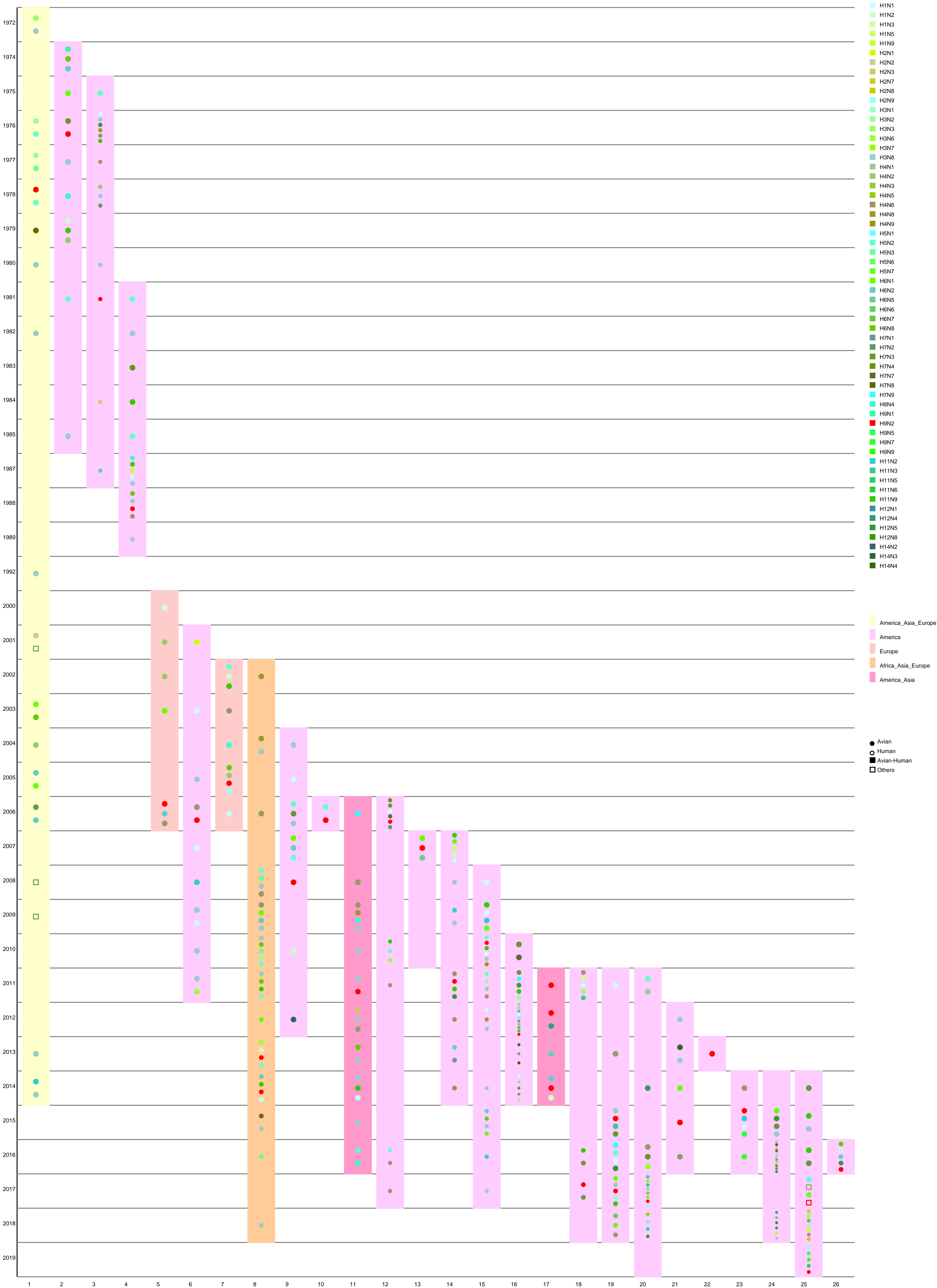

Supplement: Supplementary Figure 6 — Complete information about typical clusters of NS. [file Image_6.PDF]
