# Supplementary material for: Characterizing the Core Internal Gene Pool of H9N2 Responsible for Continuous Reassortment With Other Influenza A Viruses
Source: Front Microbiol. 2021 Dec 16;12:751142. doi: 10.3389/fmicb.2021.751142 (PMC8717948; doi:10.3389/fmicb.2021.751142)
Supplement: Supplementary Table 1 — Complete relationship between mutation sites and geographic distribution of PB2. All mutation information was obtained by comparing the filtered sequence data. [file Table_1.PDF]

Supplementary Table 1.

Location	Mutation site
America	477I，647M，452Q，291M，450L
Asia	388K，647V，273T，587V，196N，597V，675V，423S，105A，291V
Europe	18V

Supplementary Table 2

Location	Mutation site
America	58S
Asia	256A，572A，367V，47K，524V，13V，382K，386E，429K，627M，637D，371I，681V，171D，112I
Europe	154I

Supplementary Table 3

Location	Mutation site
America	266S, 544V, 215N, 399Q, 271E
Asia	408N, 60T, 404C, 336T, 422V, 625N, 62I, 36S, 261R, 715R, 606L, 271N, 387N, 355R, 342S, 69V, 683E, 553V
Africa	516M, 321T

Supplementary Table 4

Location	Mutation site
America	449N, 304K, 495F
Asia	185I, 372A, 351M, 238V, 376N, 51N, 94Q, 76R, 216V, 104V, 33S

Supplementary Table 5

Location	Mutation site
America	206G
Asia	36A, 143L, 156A, 166A, 191V, 94K, 58V, 106M, 233I, 245L, 100K, 165A, 53S, 14I, 138N

Supplementary Table 6

Location	Mutation site
America	128T, 162M, 14I
Asia	12G, 118I
